# The Association Between Area Deprivation Index and Patient-Reported Outcomes in Patients with Advanced Cancer

**DOI:** 10.1089/heq.2020.0037

**Published:** 2021-01-19

**Authors:** Margaret Quinn Rosenzweig, Andrew D. Althouse, Lindsay Sabik, Robert Arnold, Edward Chu, Thomas J. Smith, Kenneth Smith, Douglas White, Yael Schenker

**Affiliations:** ^1^ Department of Acute and Tertiary Care, School of Nursing, University of Pittsburgh, Pittsburgh, Pennsylvania, USA.; ^2^ Center for Research on Health Care Data Center, School of Medicine, University of Pittsburgh, Pittsburgh, Pennsylvania, USA.; ^3^ Department of Health Policy and Management, Graduate School of Public Health, University of Pittsburgh, Pittsburgh, Pennsylvania, USA.; ^4^ Division of General lnternal Medicine, School of Medicine, University of Pittsburgh, Pittsburgh, Pennsylvania, USA.; ^5^ Division of Hematology/Oncology, School of Medicine, University of Pittsburgh, Pittsburgh, Pennsylvania, USA.; ^6^ Harry J. Duffey Family Professor of Palliative Medicine, Sidney Kimmel Comprehensive Cancer Center, Johns Hopkins University, Baltimore, Maryland, USA.; ^7^ Department of Critical Care Medicine, School of Medicine, University of Pittsburgh, Pittsburgh, Pennsylvania, USA.

**Keywords:** advanced cancer, neighborhood deprivation index, anxiety, low deprivation

## Abstract

**Background:** This analysis describes associations between area deprivation and patient-reported outcomes among patients with advanced cancer.

**Methods:** This is a cross-sectional analysis of baseline data from a multisite primary palliative care intervention trial. Participants were adult patients with advanced cancer. Patient-level area deprivation scores were calculated using the Area Deprivation Index (ADI). Quality of life and symptom burden were measured. Uni- and multivariate regressions estimated associations between area deprivation and outcomes of interest.

**Results:** Among 672 patients, ∼0.5 (54%) were women and most (94%) were Caucasian. Mean age was 69.3±10.2 years. Lung (36%), breast (13%), and colon (10%) were the most common malignancies. Mean ADI was 64.0, scale of 1 (low)–100 (high). In unadjusted univariate analysis, Functional Assessment of Cancer Therapy—Palliative (*p*=0.002), Edmonton Symptom Assessment Scale (*p*=0.025) and the Hospital Anxiety and Depression Scale anxiety (*p*=0.003) and depression (*p*=0.029) scores were significantly associated with residence in more deprived areas (*p*=0.003). In multivariate analysis, controlling for patient-level factors, living in more deprived areas was associated with more anxiety (*p*=0.019).

**Conclusion:** Higher ADI was associated with higher levels of anxiety among patients with advanced cancer. Geographic information could assist clinicians with providing geographically influenced social support strategies.

## Introduction

The influence of income on cancer care outcomes is well-recognized.^1–5^ In recent years, an expanded view of social determinants of health (SDOH) includes not only individual or family income, but also the impact of area or “place” and its associated deprivation on individual health and wellbeing in cancer.^6^ The measurement of area-level social and economic deprivation in both rural and urban communities can serve as a predictor of a more global perspective of health and cancer care outcomes than the conventional metric of individual and family income data.^7,8^ To date, the association between area-level deprivation and patient outcomes in advanced cancer has not been explored.

Research describing socioeconomic or racial disparities in cancer outcomes has usually focused on a narrow range of outcomes, such as disease progression and survival. These disparities are often explained through inequity in access to care for screening and treatment.^8,9^

Inclusion of a broader range of SDOH such as area or “place” considerations into the explanatory framework for outcome disparities expands the hypothesized, mechanistic process. These additional considerations beyond simply “access to care” include more specific, immediate consequences associated with area deprivation and cancer care, such as disparity in patient perceived financial toxicity,^10–12^ access to symptom management medications,^13–15^ poor overall quality of life,^16^ and social isolation^17^ that may ultimately influence patient reported outcomes, disease progression and survival.

Although area/neighborhood and community are sometimes used interchangeably, the constructs are distinctive. While area or neighborhood represents a specific area of belonging, an individual may concurrently consider themselves to be a member of many communities incorporating population, religion, or ethnicity.^18^ Both are important, but because the measurement of the area influence and associated deprivation can provide evidence regarding the need for targeting of specific area-level interventions, area or neighborhood is the focus of our analysis.

The Area Deprivation Index (ADI) is a measure of socioeconomic neighborhood deprivation. The ADI encompasses a composite of several neighborhood characteristics, such as poverty, housing, employment, and education.^19–21^ In prior work, neighborhood-level deprivation measured using the ADI has been associated with higher overall mortality among patients with head and neck, lung, and prostate cancer.^2,22,23^

To date, research examining the potential association between area deprivation and patient-reported outcomes including quality of life, symptom burden, and symptoms of anxiety and depression among patients with advanced cancer is lacking. This is an important gap in the literature as understanding this association between area deprivation and patient-reported outcomes can inform targeted, neighborhood-level interventions for patients near the end of life.

The goal of this study was to investigate the association between area deprivation and patient reported outcomes including symptom burden, quality of life, and symptoms of depression and anxiety reported by patients with advanced cancers. We hypothesized that patients from areas with greater deprivation would report a higher incidence and severity of symptoms, worse quality of life, and more symptoms of depression and anxiety.

## Methods

### Design

This descriptive and correlative analysis utilized baseline data from a cluster randomized trial of a primary palliative care intervention entitled CONNECT (Care management by Oncology Nurses to Address Supportive care needs; National Cancer Institute 1R01CA197103). This study was approved by the University of Pittsburgh Institutional Review Board (PRO15120154) and registered on clinicaltrials.gov (NCT02712229). The specific details of the study design have been published previously.^24^ All measures for this analysis were collected before intervention delivery.

### Setting and participants

This study was conducted in 17 oncology practices in Western Pennsylvania. All patients completed a written informed consent process. Eligible patients were 21 years or older with metastatic solid tumor cancers for whom the oncologist “would not be surprised if the patient died within 1 year.”^25^ In addition, only patients with an Eastern Cooperative Oncology Group (ECOG) performance status of 0 (full, normal activity), 1 (some restriction in physical activity) or 2 (limited in work activities) were eligible.^26^ Patients with poor performance status (ECOG 3 or 4), poor cognition, and/or inability to understand English were excluded. Inclusion criteria included the requirement that participants anticipated receiving oncology care at the site of recruitment. Patients were excluded if they were unable to speak or read English, unable to consent to treatment, or had hematologic malignancies. All patients signed informed consent before participating. The study was approved by the University of Pittsburgh Institutional Review Board.

### Area deprivation

Area deprivation was measured using the ADI. Based on a measure developed by the Health Resources and Services Administration over 20 years ago, the ADI allows for rankings of neighborhood by socioeconomic status deprivation, a composite of several factors including income, education, employment, and housing quality. Scores range from 0 to 100 (higher scores=higher deprivation). The ADI was derived for each participant by entering the participant's home zip code into a publicly available interactive website.^6,27,28^ From these zip codes, we abstracted median household income per zip code rather than collecting individual patient data. Zip code also provided information regarding area rurality, defined by population density.^29^

### Patient-reported outcomes

Quality of life was measured using the Functional Assessment of Cancer Therapy—Palliative (FACIT-Pal), comprising physical, social, emotional, functional, and palliative subscales (total FACIT-Pal range 0–184; higher scores reflect better quality of life).^30^ The palliative subscale includes questions related to symptoms, family/friend relationships, life closure, feeling hopeful, decision-making, and communication at the end of life.^31^

Patient symptom burden was measured using the Edmonton Symptom Assessment Scale (ESAS).^32^ Commonly used in advanced cancer, the ESAS includes nine self-reported symptoms: pain, activity, nausea, depression, anxiety, drowsiness, appetite, sense of well-being, and dyspnea. Total scores range from 0 to 90, with higher scores indicating more symptom burden.^33^

Symptoms of anxiety and depression were measured using the Hospital Anxiety and Depression Scale (HADS). The HADS are two self-administered scales that are well validated in medical populations, including patients with advanced cancer.^34,35^ Scales measure general symptoms of anxiety (HADS-A—7 items, range 0–21) and depression (HADS-D—7 items, range 0–21), with higher scores representing greater degrees of anxiety and depression symptoms, respectively.^35^

### Additional patient-level demographic and clinical factors

Patient-level variables assessed using baseline questionnaires included gender (male, female), age, race (Caucasian/white, African American/black, Asian, other), current marital status (never married, married, widowed, divorced/separated, declined to answer), current living situation (own home, renting, assisted living facility, nursing home), and how well one manages on current household income (cannot make ends meet, just manage to get by, have enough with a little extra, money is not a problem). ECOG performance status was documented by clinicians and captured as 0 (full, normal activity), 1 (some restriction in physical activity), or 2 (limited in work activities).^26^ Scores were provided by treating oncologist.

### Analysis

This report descriptively explores the independent and dependent variables, and then describes the associations between area deprivation and patient reported outcomes of quality of life, symptom burden, and symptoms of anxiety and depression for patients with advanced cancer near the end of life. Descriptive statistics including mean, median, and standard deviation are presented for the ADI, patient-reported outcomes of quality of life, symptom scores and anxiety and depression scores. Relationships between the ADI and each of the respective measures were presented visually in scatterplots; univariable and multivariable linear regression models were used to estimate associations between area deprivation and outcomes of interest.

Multivariable analyses controlled for individual-level factors identified *a priori* as associated with area deprivation and/or patient-reported outcomes, based on prior literature and expert clinical opinion. These covariates were gender,^36^ age,^36^ race,^37^ education, marital status,^38^ living situation,^39^ management on income,^37^ time since diagnosis, and active chemotherapy treatment. All statistical analyses were performed using SAS version 9.4 (SAS Institute, Cary, NC). Level of significance was 0.5. Missing data were rare at <1% of the study cohort.

## Results

The cohort was largely Caucasian (94%), older (mean age 69.3±10.2), and almost equally divided by sex (54% women; Table 1). Most were married (57%), retired (64%), and owned their own home (79%). The most common cancers were lung (36%), breast (13%), and colon (10%). At the time of enrollment, most patients reported that they were being treated with chemotherapy (69%). Performance status was largely good, with 58.5% of the sample categorized as ECOG performance status 1—defined as “restricted in physically strenuous activity but ambulatory and able to carry out work of a light or sedentary nature.” When asked about financial adequacy, patients were closely divided between “just manage to get by” (33.6%) and “I have enough with a little extra” (37.2%). Only 7% of this cohort reported they were “not making ends meet.”

**Table 1.** Demographic and Clinical Characteristics of 672 Participants

	Full trial population (*N*=672)
Age, years (mean±SD)	69.3±10.2
Sex, *n* (%)	
Male	312 (46.4)
Female	360 (53.6)
Race, *n* (%)	
Caucasian/white	632 (94.0)
African American/black	33 (4.9)
Asian	5 (0.7)
Other	2 (0.3)
Current marital status, *n* (%)	
Never married	44 (6.5)
Married	382 (56.8)
Widowed	132 (19.6)
Divorced/separated	107 (15.9)
Declined to answer	2 (0.3)
Current living situation, *n* (%)	
In a home I own	532 (79.2)
In a home I rent	106 (15.8)
In an assisted living facility	6 (0.9)
In a nursing home	3 (0.4)
Declined to answer	2 (0.3)
Other	16 (2.4)
How well are you able to manage on your income?, *n* (%)	
Cannot make ends meet	46 (6.8)
Just manage to get by	226 (33.6)
Have enough with a little extra	250 (37.2)
Money is not a problem	108 (16.1)
Declined to answer	41 (6.1)
ECOG Eastern Co-operative Oncology Group, *n* (%)	
Fully active	157 (23.4)
Restricted in physically strenuous activity but ambulatory and able to carry out work of a light or sedentary nature	393 (58.5)
Ambulatory and capable of all self care but unable to carry out any work activities. Up and about more than 50% of waking hours	122 (18.2)

ECOG, Eastern Cooperative Oncology Group; SD, standard deviation.

The mean ADI was 64.2 (standard deviation [SD]=23.0). Utilizing these zip codes, it was determined that the majority, 65.5% of patients lived in counties classified as low income and 49% were designated to be rural by population density of <500 residents/square mile. By zip code analysis, the median household income was $56,801. Among the low-income zip codes, the median household income was $46,893.

The baseline distribution of patient-reported outcomes is summarized in Table 2. The mean FACIT-Pal score was 130 (SD=25.5). These scores were consistent with previously reported scores among patients with advanced cancer of 132 (SD=24.7).^35^ Composite symptom score as measured by ESAS was consistent with other populations with advanced cancer (mean total ESAS score 25.2, SD=16). Among patients with advanced cancer, mean ESAS scores are historically reported in a range of 20.7–27.2 (Refs.^40^). Mean HADS scores for the anxiety (5.78, SD=3.9) and depression (5.4, SD=3.75) subscales indicated mild overall symptoms of anxiety and depression. In advanced cancer, scores across several studies were 8.1 (SD=3.9) to 8.56 (SD=3.71) for HADS-A and 6.0 (SD=4.2) to 9.82 (SD=4.2) for HADS-D.^36,37^

**Table 2.** Baseline Distribution of Patient-Reported Outcomes

FACIT-Pal	
Total score	130±25.5
ESAS	
Total score	25.2±16.0
HADS total scores	
Depression	5.41±3.75
Anxiety	5.78±3.90

ESAS, Edmonton Symptom Assessment Scale; FACIT-Pal, Functional Assessment of Cancer Therapy—Palliative; HADS, Hospital Anxiety and Depression Scale.

**FIG. 1.**
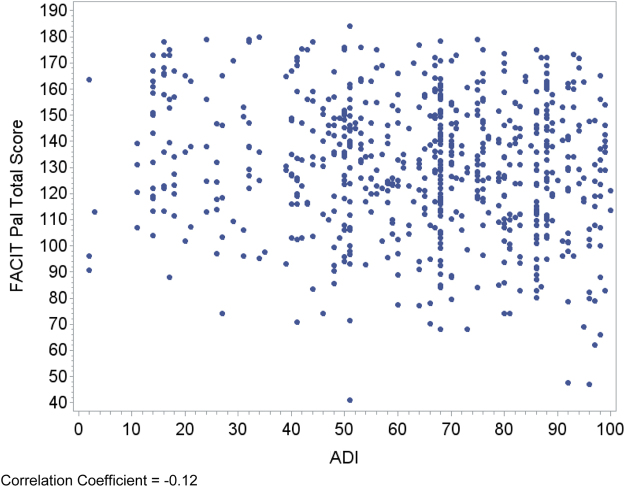
Correlation coefficient=−0.12. FACIT-Pal, Functional Assessment of Cancer Therapy—Palliative; ADI, Area Deprivation Index.

**FIG. 2.**
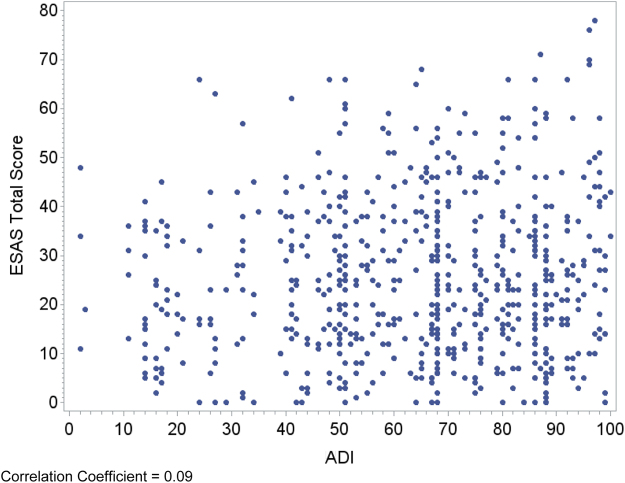
Correlation coefficient=0.09. ESAS, Edmonton Symptom Assessment Scale.

**FIG. 3.**
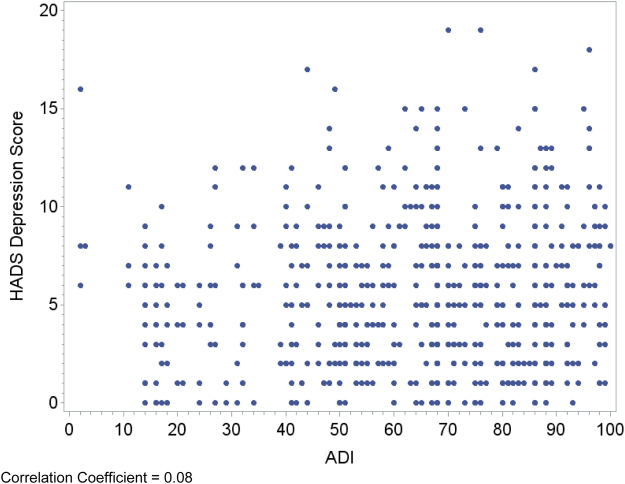
Correlation coefficient=0.08. HADS, Hospital Anxiety and Depression Scale.

**FIG. 4.**
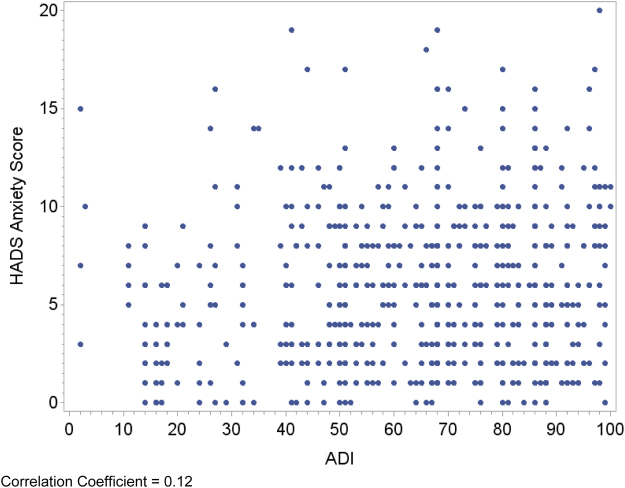
Correlation coefficient=0.12.

Relationships between area deprivation and the respective patient outcomes are plotted visually (Figs. 1–4) with regression analyses reported in Table 3. In an unadjusted analysis, higher area deprivation was associated with worse quality of life (*p*=0.002). An increase of 10 points in ADI was associated with 1.33 lower score on baseline FACIT-Pal (95% confidence interval=−2.16 to −0.50). Higher area deprivation was also associated with worse symptom burden (*p*=0.025) and worse symptoms of depression (*p*=0.029) and anxiety (*p*=0.003).

**Table 3.** Unadjusted and Adjusted Associations Between Neighborhood Deprivation and Patient-Reported Outcomes

	β^*^	Unadjusted	*p*	β^*^	Adjusted^**^	*p*
		95% CI			95% CI	
FACIT-Pal						
Total score	−1.33	(−2.16 to −0.50)	0.002	−0.73	(−1.59 to 0.13)	0.097
ESAS						
Total score	0.60	(0.08 to 1.13)	0.025	0.18	(−0.37 to 0.73)	0.526
HADS						
Depression	0.14	(0.01 to 0.26)	0.029	0.10	(−0.03 to 0.23)	0.148
Anxiety	0.20	(0.07 to 0.33)	0.003	0.16	(0.03 to 0.30)	0.019

^*^ Coefficients have been scaled to reflect the change in expected value of each item for a “10 point” increase in ADI (because ADI is a 0–100 scale, reporting the change in expected value of each outcome per “1 point” change in ADI is difficult to interpret).

^**^ Adjusted model reports the relationship between ADI and patient-reported outcome with covariate adjustment for gender, age, race, marital status, living situation, how well they can manage on income, and ECOG Performance Status.

CI, confidence interval; ADI, Area Deprivation Index.

In multivariable analyses controlling for individual factors associated with patient-reported outcomes, higher area deprivation remained significantly associated with increased anxiety symptoms (*p*=0.019). However, the relationships were attenuated for the effect of area deprivation on quality of life (*p*=0.097) and depression symptoms (*p*=0.148). Multivariable adjustment removed the effect of area deprivation on symptoms (*p*=0.526).

## Discussion/Conclusions

Among a cohort of largely Caucasian, married patients with advanced cancer in Western Pennsylvania, we found associations between area deprivation and patient-reported outcomes. Higher area deprivation remained independently associated with more anxiety symptoms after controlling for patient-level demographic and clinical characteristics. In other areas of health care, higher areas of deprivation are associated with worse outcomes ranging from infant mortality^41^ to higher 30-day readmission risks,^42^ chronic disease burden,^43^ and overall life expectancy.^44^

Although acknowledged as potentially important for health outcomes, the influence of neighborhood has not been previously well examined in studies on cancer care outcomes beyond survival. These findings support the concept that residing in more deprived neighborhoods may influence patient-reported outcomes among patients with advanced cancer. In addition, these findings suggest that measures of patient-reported outcomes such as anxiety may be reflective of social deprivation in more deprived areas, providing important targets for clinical intervention.^45,46^ Owing to the secondary analysis of this data set the variables that may be more profoundly influenced by deprived neighborhoods such as neighborhood institutions and resources including access to physicians and pharmacies, physical stressors in the neighborhood, or fear for personal safety were not measured in the analysis.^47^

It is particularly intriguing that anxiety symptoms remained significantly worse for patients from more deprived neighborhoods after controlling for individual patient factors thought to influence these relationships. It is well established that individuals from lower income households experience more cumulative lifetime stress such as chronic food and housing insecurity, fear for personal safety, and exposure to violence over a lifetime with a “weathering” effect that holds implication for health outcomes.^48^

For advanced illness the Model of Neighborhood Material Wealth outlined by Stafford and Marmot^49^ asserts that regardless of individual factors, neighborhoods of less deprivation (wealthier) offer their residents more tangible goods and services decreasing the stress of “everyday life” associated with advanced illness. Examples of these goods and services in advanced cancer care may be access to pharmacies with needed supplies and medications, more and better funded social support through faith-based and community groups, or supplementary patient-based home support (cleaning, child care, and transportation) through advocacy agencies and individuals within the community able to volunteer and offer support.

Instead of traditionally focusing on individual-level intervention for mitigation of income-level disparities in cancer care outcomes, strengthening the individual community through collaborations with established agencies to assist with such services have proven to be helpful in other advanced illness. Pesut et al. piloted an intriguing community-based program linking trained palliative care volunteer navigators to patients and families in underserved communities.^50^ The CAPABLE Project successfully combines handyman, nursing, and occupational therapy services to homebound elderly to preserve aging in place and limit hospital readmission. This pragmatic, community-based program could be applied to patients with advanced cancer, potentially limiting individual-level anxiety.

Within the cancer clinics, assessment and mitigation of issues related to SDOH can now be billable using International Statistical Classification of Diseases codes in the form of “Z-codes” allowing strategies for the implementation of assessment, tracking, and mitigation of SDOH that may adversely affect health outcomes among specific populations.^51,52^ Additional strategies to assist patients with advanced cancer at the neighborhood level may involve patient navigation, traditionally utilized to assist with screening and appointment adherence, provided by community health workers familiar with the local resources for patients with advanced illness.^53^ This community navigation role has recently been expanded to work with patients toward advanced directives^54^ and could be considered in a broader, more supportive role for patients and families coping with advanced illness.

An examination of the influence of area deprivation may provide foundational information for clinical and community care providers to include “place” in the risk profile for patients at the end of life. This secondary examination, although limited in depth and breadth for assessment of neighborhood variables important to discern neighborhood support, points to a need for more proactive assessment regarding the status of the patient's community. Patients with advanced cancer may be provided with screening on first visit for important SDOH in a standardized manner and then have appropriate referral according to specific need.^55,56^ For example, with the patient's permission, an immediate and proactive referral to emergency food services for patients with food insecurity or link to community or faith communities for available home and emotional support could be provided.

Although the findings from this secondary analysis are intriguing, our study has several limitations, which limits the discussion of area influence on patient-reported outcomes beyond this exploratory analysis. First, the study was exploratory without *a priori* hypothesis. The cross-sectional design does not allow for an exploration of the impact of area or neighborhood across the advanced cancer trajectory. The demographics of the patients in these cancer clinics are relatively homogenous, limiting the exploration of other important SDOH. An additional limitation was that income was not collected at the individual level and needed to be calculated from patient zip code.

This secondary analysis did not include questionnaires for fully exploring factors specifically related to area deprivation, particularly relevant for the further exploration of stress, such as the impact of social isolation, access to medication, providers, transportation, and neighborhood belief system on outcomes related to advanced cancer. Specific areas of deprivation would provide direction for further descriptive or beginning interventional work.

These findings and recognition of limitations do inform planning for future studies. A future study focusing on neighborhood deprivation and advanced stage cancer should incorporate a scan of neighborhood resources specific to patients with advanced stage cancer to determine if the presence of goods and services impacts relevant patient-reported outcomes. Assessment of the individual could be expanded to include the patient's lifetime exposure to stress, patient's perceptions of the neighborhood status including the neighborhood barriers to care, the collective financial strength of the neighborhood, satisfaction with neighborhood services, and the patient's perception of their social status as the result of living in their neighborhood. In addition, the assessment of neighborhood deprivation could be expanded to incorporate more than the ADI with factors specific and critical to care for patients with advanced cancer.

Understanding the role of area deprivation on patient reported outcomes in advanced cancer can be important, incorporated into clinical practice, and may inform targeted interventions at the provider, system, and policy levels that can improve patient outcomes. Future research focusing on implementation and assessment strategies are important next steps.

## Abbreviations Used

ADI: Area Deprivation Index
CI: confidence interval
ECOG: Eastern Cooperative Oncology Group
ESAS: Edmonton Symptom Assessment Scale
FACIT-Pal: Functional Assessment of Cancer Therapy-Palliative
HADS: Hospital Anxiety and Depression Scale
HADS-A: HADS—Anxiety
HADS-D: HADS—Depression
SD: standard deviation
SDOH: social determinants of health
